# Beef-on-dairy calf management practices in commercial calf ranches

**DOI:** 10.1093/tas/txaf064

**Published:** 2025-05-08

**Authors:** Rebecca A Bigelow, Phillip A Lancaster, Brad J White, Raghavendra G Amachawadi, Tera R Barnhardt, Miles E Theurer

**Affiliations:** Beef Cattle Institute, Department of Clinical Sciences, College of Veterinary Medicine, Kansas State University, Manhattan, KS 66506, USA; Beef Cattle Institute, Department of Clinical Sciences, College of Veterinary Medicine, Kansas State University, Manhattan, KS 66506, USA; Beef Cattle Institute, Department of Clinical Sciences, College of Veterinary Medicine, Kansas State University, Manhattan, KS 66506, USA; Beef Cattle Institute, Department of Clinical Sciences, College of Veterinary Medicine, Kansas State University, Manhattan, KS 66506, USA; Heritage Vet Partners, Johnson, KS 67855, USA; Veterinary Research and Consulting Services, Hays, KS 67601, USA

**Keywords:** calf raising, calf starter feed, calf weaning, health protocol, milk feeding protocol, survey

## Abstract

The number of beef-on-dairy calves being produced has been steadily increasing. Many calves are sent off-site to calf ranches for raising after birth. The objective of this survey was to describe management practices of beef-on-dairy calves in commercial calf ranches. A total of 15 calf ranches were surveyed in 3 regions: the High Plains (n = 7), Midwest (n = 6), and West (n = 2). Operation capacities were categorized as less than 1,000, between 1,000 and 20,000, 20,000 to 50,000, and greater than 50,000 calves. All operations received calves less than 4 d of age. There was a variety of types of pre-weaning housing. Almost all operations fed milk replacer with one operation feeding saleable milk; feeding protocols (timing and quantity) varied among operations. Every operation offered calf starter upon arrival, but formulation of starter differed among ranches. Weaning age ranged between 42 and 72 d with about 53% of operations weaning calves at 60 d or greater. Calves spent anywhere between 0 and 180 d in a group pen setting. Thirty-three percent of operations moved calves through multiple group pens post-weaning. Similarly, 33% of operations transitioned calves through multiple diets once they were in group pens. Most operations fed the transition/grower diet ad libitum, however the ingredients used were variable among operations. All operations administered at least 2 health products such as vaccines, antimicrobials, etc. while the calf was on the property. These results provide important information regarding the management of beef-on-dairy calves at commercial calf ranches.

## Teaser

While there is literature describing how dairy replacement heifers are raised, there is little information published on practices for raising beef-on-dairy cross calves on calf ranches. This survey describes the practices used by calf ranches that raise beef-on-dairy cross calves in the High Plains, Midwest, and Western US.

## INTRODUCTION

In recent years, the use of semen from beef sires has become increasingly common within the dairy industry to produce beef-on-dairy calves. From 2022 to 2023, the National Association of American Breeders reported a shift in semen sales to an increased number of units of beef semen, 1.5 million units, sold while dairy semen sales decreased by 2.5 million units, indicating a shift in breeding practices within the dairy industry (Weiker, 2024). Separating calves from their dams as young as 1 d of age is a common practice within the dairy industry. In 2007, the USDA National Animal Health Monitoring System (NAHMS) survey reported that about 1 in 10 operations raised some dairy heifers off the operation, and by 2014, approximately one-fourth of all heifer calves were raised off-site (USDA, 2010, 2021). These off-site operations are often termed calf ranches or calf raising facilities. A calf ranch can be defined as an operation that raises calves from a young age to a specific weight or age dependent on the production plan for those animals.

Previous literature has reported management practices that are implemented at preweaned replacement heifer raising facilities (USDA, 2010, 2012, 2021; Urie et al., 2018). Additionally, there have been surveys conducted with beef cow-calf producers to record herd management practices in the U.S. and Canada (USDA, 1997; Hanzlicek et al., 2013; Woolums et al., 2013; Murray et al., 2016). However, little to no information is published regarding the management of beef-on-dairy cross calves in commercial calf ranches. In 2007, Walker et al. (2012) conducted a cross-sectional study with calf ranches that exclusively reared heifers and operations that raised a mix of heifer and bull calves. Of all the preweaned calves raised on the mixed operations, a median of 73% were heifers and 23% were bulls (Walker et al., 2012). However, there was no specification as to the breed of the bull calves being raised. Machado and Ballou (2022) completed a literature review to summarize the common management practices associated with calf raising facilities in the U.S.; however, the authors highlighted the lack of knowledge surrounding management of beef-on-dairy calves and advocated for more research to be completed in this area. There have been surveys conducted with replacement heifer raising facilities, however, there has only been speculation that beef-on-dairy calves on the same operations may be managed differently. Therefore, the objective of this study was to describe common management practices of beef-on-dairy calves implemented on commercial calf ranches.

## MATERIALS AND METHODS

This survey (#IRB-11903) was reviewed by the Institutional Review Board (IRB) for Kansas State University and deemed exempt from further review. The survey was designed by the authors in discussion with commercial calf ranch owners, managers, and consulting veterinarians. Participants were identified through personal communication with consulting veterinarians. Due to this approach, the sampling can be described as a convenience sample. This resulted in a small sample size of 15 calf ranches. A comprehensive survey was developed to collect information regarding the management practices implemented at commercial calf ranches feeding beef-on-dairy calves. The survey was focused on management of beef-on-dairy calves and consisted of 74 questions. To be included in the research, the operation had to raise beef-on-dairy calves in some proportion. For operations that raised both dairy and beef-on-dairy calves, data was not collected on management of the dairy animals. There were 10 sections of the survey including general information/ranch demographics, calf arrival processing procedures, pre-weaning housing, milk feeding protocol, starter feed formulation and feeding protocol, water offerings, weaning protocol, movement/management of group pens, transition/grower diet formulation and feeding protocol, and health challenges and vaccine/treatment protocols. The participants were not required to answer any questions that they were not comfortable with or if they had proprietary information/protocols that could not be shared.

For general information/ranch demographics, there were 8 questions. One multiple-choice question pertained to whether the calf ranch, dairy, or a combination of the two owned the calves being raised. This section also included questions about the participant interviewed, the capacity of the ranch, and population demographics related to the proportion of beef-on-dairy and dairy replacement heifers raised. The section about calf arrival processing consisted of 11 questions. Six of the questions were about the age of the calves, number received, frequency of receival, and regions from which the operations received calves. When asked about where they receive calves from, each operation was provided with a map that was used in the “Health Management on U.S. Feedlots 2021 Phase 1 Questionnaire” (USDA-APHIS, 2020). The remaining 5 questions asked about the specific practices performed on all calves upon arrival at the ranch including tagging, castration, metaphylaxis, etc. The section for pre-weaning phase housing consisted of 8 questions, 6 of which were multipart. First, the participants were asked if they housed preweaned calves in group pens or individually. Then, depending on that answer, they were directed to multipart questions describing the housing. The participants were asked about housing calves indoors/outdoors, in hutches or individual pens, specific characteristics about the housing such as if hutches/pens were elevated, total square footage, distance between hutches/pens, and whether the ranch provided bedding or not.

The milk feeding portion had 14 questions. When asked about type of milk fed, the participants could choose up to 3 choices: milk replacer, nonsaleable/discount milk, saleable milk. If they chose more than one type, they were asked to report the percentage of each type they fed. They were also asked about formulation of milk replacers, pasteurization protocols, target percent protein and fat, the addition of anything to the milk such as vitamins, minerals or antibiotics, their milk feeding protocol and the method of feeding. Questions about milk feeding protocol were structured into a table where the participants were asked how many times calves were fed per day, quantity fed, and how those might change as the calf aged. Similarly, there was a table for starter feed for the surveyor (RB) to record the ration type (pelletized, total mixed ration (TMR), texturized feed), the times fed per day, the quantity fed and how that may change as the calf aged. This section also consisted of close-ended questions regarding the formulation of the starter diet. These included whether the starter feed included roughages, grains, liquids, and/or feed additives, and if so, what kind. The section about water consisted of 5 questions about when water was first offered, how it was offered, how often it was changed out, frequency of thawing ice in the winter, and frequency of cleaning the buckets/troughs/drinkers.

The weaning section consisted of 7 questions related to weaning age of calves, reasons calves may be weaned earlier/later, criteria used to make weaning decisions, age calves are moved to group pens, and reasons calves may be moved earlier or later to group pens. The first question of the group pen section was structured into a table where the surveyor (RB) recorded the number of different group pens that calves were moved through, the number of head per pen, the size/dimensions of the pen, and the linear bunk space. The remaining 7 questions were related to pen environment such as bedding, enrichment, cleaning of the pens, and cleaning of the bunks and waterers. For the transition/grower feed, there was another table to record the ration type, number of times fed per day, and quantity fed in relation to age of the calf. Similar to the starter feed section, the participants were asked about the formulation of the diet and what ingredients were used.

For health, participants were first asked about measuring serum total protein. Next, they were asked about their vaccine/health protocol. The 6 calf ranches from the Midwest all used the same consulting veterinarian, so for the health section, those participants’ answers were counted as 1 observation due to the fact they had the same health protocol. The participants were not required to give the specific vaccines used, only the type of vaccines and at what age the vaccines were given. For example, the surveyor (RB) listed off the different kinds such as viral respiratory, bacterial respiratory, autogenous, clostridial, scours, or other. Next, questions were asked about respiratory and digestive diseases. Participants were asked about treatment protocols for each disease listing only the type of product such as an antimicrobial, anti-inflammatory, electrolytes, or other products. They were then asked about precautions taken in adverse weather events such as winter storms or heat waves. Finally, they were asked about whether they kept individual treatment records.

Between November 2023 and June 2024, surveys were conducted in-person (n = 13) or face-to-face via video call on Microsoft Teams (n = 2). For the in-person visits, the participant would give the surveyor (RB) a tour of the facility while answering questions followed by sitting down to verify that all questions were answered. The survey answers were then entered into a Microsoft Form upon return to Kansas State University. Data cleaning and summarization was performed using Microsoft Excel and pivot tables. All questions were answered by every operation unless the question was not applicable (N/A). For example, one operation shipped calves to a different location immediately following weaning, so any questions about post-weaning housing and feed were N/A. Categorization of answers was completed post hoc. The surveyor examined every question and the answers to determine whether answers could be summarized when appropriate. For example, total capacities were summarized as less than 1,000, 1,000 to 50,000, and greater than 50,000. Frequencies of participants’ answers were calculated as a percentage of operations for each question to describe the management practices used at calf ranches.

## RESULTS

### General Information/Ranch Demographics

The calf ranches were located in Kansas (n = 4), Texas (n = 2), New Mexico (n = 1), Indiana (n = 4), Ohio (n = 2), and California (n = 2). The ranches from Kansas, Texas and New Mexico were categorized as the High Plains region. The ranches from Indiana and Ohio were grouped into the Midwest region, and the California ranches were classified as the West region. In total, 15 calf ranches were surveyed with 47% (7/15) residing in the High Plains region, 40% (6/15) from the Midwest, and 13% (2/15) from the West. Of the surveys, 2 were conducted with the operation’s veterinarian, 4 were with the general manager, and 9 were conducted with the owner of the operation. The operations were classified as either a calf ranch only (12/15; 80%) or a combination of a calf ranch and dairy (3/15; 20%). None of the calf ranches had sole ownership of the calves, as 60% of the operations reported the dairies where calves were born/received from retained ownership and 40% reported a combination of the dairies and calf ranch owning the calves. Size of operation was categorized with 40% (6/15) of ranches having a capacity of less than 1,000 calves, 13% (2/15) between 1,000 and 20,000, 20% (3/15) between 20,000 to 50,000, and 27% (4/15) having a capacity greater than 50,000 calves. All ranches that had a capacity of 1,000 calves or less exclusively raised beef-on-dairy calves. Operations between 1,000 to 20,000 calves raised a proportion of beef-on-dairy calves between 50 and 70% of their total capacity, and those that had a capacity between 20,000 and 50,000 raised between 14 and 100% beef-on-dairy. Operations that were greater than 50,000 head raised a proportion of beef-on-dairy calves between 35 and 70% of their total capacity.

### Calf Arrival Processing Procedures

Most of the operations (67%) received calves when they were 2 to 4 d of age, and 33% received calves that were 0 to 1 d of age. The number of calves received at one time varied from less than 100 to greater than 250 calves with 33% receiving less than 100, 33% receiving between 100 and 250, and the final 33% receiving greater than 250 calves at one time. For frequency of receiving calves, 47% received new loads of calves daily, 13% received loads once to twice a week, and 40% received loads every 5 wk or longer. The 40% that received loads every 5 wk or longer were all located in the Midwest region. When asked if frequency of receiving calves fluctuated based on season, 60% reported “Yes,” and 40% reported “No.” Of the 9 operations that reported changes based on season, 3 reported that fall was their busiest season for receiving calves, 2 winter, 1 spring, and 3 in the summer. Based on the provided map, 20% (3/15) of the operations received calves from a state in region 1, 33% (5/15) received from a state in region 2, 27% (4/15) received from a state in region 3, 60% (9/15) received from a state in region 4, 13% (2/15) received from a state in region 5, and 40% (6/15) received calves from a state in region 6. No operations received calves from region 7 or 8. Eight of the operations (53%) received calves from multiple regions. This is the reason the percentages above are greater than 100% when summed.

For arrival processing of all calves, 40% of operations reported they tag calves with their own form of identification. For castration, 47% of operations used banding as their preferred castration method, and 53% of operations performed surgical castration. Twenty percent of operations castrated calves when they were less than 7 d of age, 33% castrated calves between 7 and 14 d of age, and 47% waited until calves were greater than 14 d of age to castrate. Most operations (87%) reported that they did not feed colostrum to newly received calves. Upon arrival, 33% reported giving an antibiotic metaphylactically to all calves, 53% reported they did not, and 13% reported that they sometimes gave metaphylaxis. One operation gave metaphylaxis in the summer, and the other operation reported they would give metaphylaxis based on the visual assessment of the health of the calves upon arrival.

### Pre-Weaning Housing

Of all the operations, 6 ranches housed their pre-weaned calves indoors and 9 housed calves outdoors. All operations that housed calves indoors were located in the Midwest. Upon arrival, 60% housed calves individually in hutches, 33% housed calves in individual pens, and 7% housed calves in group pens. For the 9 operations that use hutches, 44% used elevated hutches and 56% did not. As for hutch material, 44% used plastic hutches and 56% used wooden hutches. When asked about total square footage of the hutches/pens, 47% (7/15) reported less than 15 square feet as the dimensions for the hutch/pen, 47% (7/15) reported between 15 and 50 square feet, and 7% (1/15) reported greater than 50 square feet. The operation reporting greater than 50 square feet per calf was the operation that used group pens to house pre-weaned calves. Most calves (80%) were close enough to have nose-to-nose contact with each other. For bedding, 87% reported bedding down hutches/pens. Of those 13 operations, 38% bedded seasonally in the winter and 62% bedded all year round. The material used for bedding consisted of corn stalks, shavings, or straw. Most operations (62%; 8/13) that provided bedding reported adding new bedding on top of old as needed then cleaning completely between groups of calves. Other operations changed bedding between groups of calves (23%), monthly (7.5%), or weekly (7.5%). When asked about cleaning the hutches/pens, 87% (13/15) reported cleaning them between groups of calves and 13% (2/15) reported never cleaning. Of the 13 operations that do clean their hutches/pens, 23% power-washed them, 15% disinfected them, and 62% power-washed and disinfected them.

### Milk Feeding

**Figure 1.**
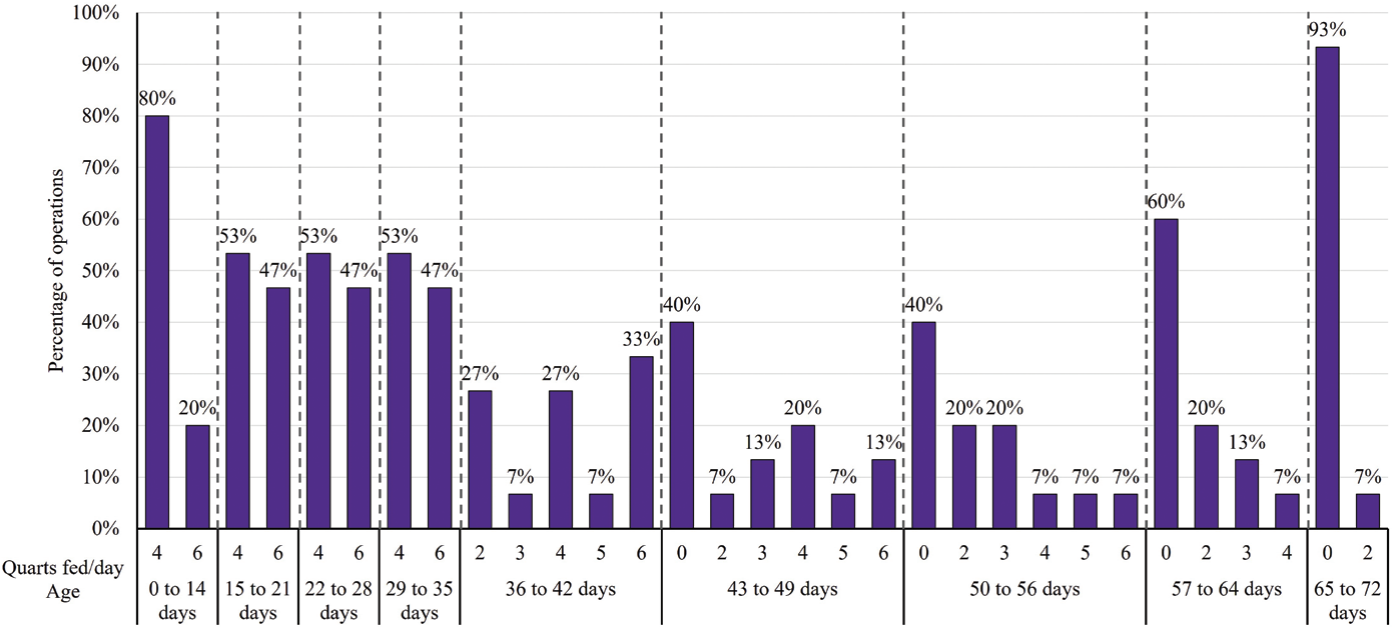
Number of quarts of milk fed per day by percentage of operation by days of age. All ranches completely weaned calves off of milk by 72 d of age.

For the first milk offering, 6 of participants offered milk upon arrival, 3 offered milk within a few hours of arrival, 2 offered milk at the first feeding following arrival, and 4 waited until the second feeding following arrival to offer milk. All participants reported that they assisted the calves with the first feeding, regardless of when the feeding took place, and 73% reported offering milk for the first feeding and the remaining 27% offered electrolytes as the first feeding. Milk feeding protocols varied based on the quantity fed to calves over time (Figure 1). The choices for type of milk fed consisted of milk replacer, nonsaleable milk, saleable milk, or a combination of these types of milk. Nonsaleable milk was defined as milk that was unable to enter the market for human consumption, sometimes described as discounted milk. Saleable milk was defined as milk safe for human consumption but did not enter the market. Of the 15 calf ranches, 47% fed milk replacer only, 27% fed nonsaleable milk in addition to milk replacer, 7% fed saleable milk in addition to milk replacer, 13% fed all three types of milk, and 7% fed only saleable milk. The operations that exclusively fed milk replacer were located in the Midwest. Of those 14 operations that fed milk replacer, 79% fed a commercial brand of milk replacer and 21% made their own milk replacer on site. For feeding nonsaleable milk, 13% (2/15) fed it to all calves, 27% (4/15) fed it exclusively to calves older than 14 d of age, and the remaining 60% (9/15) of operations did not feed nonsaleable milk. All operations that fed any proportion of nonsaleable or saleable milk pasteurized the milk prior to feeding.

Average target percent protein in the milk replacer for operations consisted of 20 (7%; 1/15), 22 (33%; 5/15), 24 (13%; 2/15), 26 (27%; 4/15), 27 (13%; 2/15) and 36 (7%; 1/15). For average target percent fat in the milk replacer, values ranged from 18 (13%; 2/15), 20 (53%; 8/15), 22 (13%; 2/15), 24 (13%; 2/15), and 30 (7%; 1/15). Of the total participating operations, 7% reported adding antibiotics to the milk, 33% added vitamins and minerals, 53% added vitamins, minerals, and antibiotics, and 7% reported no inclusion of additives to the milk. The types of milk feeders used varied among operations with 73% using bottles, 7% using buckets with a nipple on them, 7% using a milk trough, and 13% using bottles first then switching to open buckets later in the feeding period. For cleaning of bottles, 1 ranch washed the bottles with detergent between each calf and filled with disinfectant overnight, 6 rinsed the bottles with water between each calf and disinfected between each feeding period (after morning feeding and after evening feeding), 5 rinsed and disinfected bottles between each feeding period, 1 rinsed and disinfected between each calf, and 2 did not feed with bottles and therefore did not answer this question. For nipples, 1 operation washed them between calves and washed them twice before each feeding for calves under 20 d of age, 1disinfected nipples between each calf, 2 rinsed them between each calf and disinfected them between morning and evening feedings, 4 rinsed and disinfected them between each calf, 6 rinsed and disinfected between each feeding, and 1did not answer as they did not use nipples for feeding as they fed milk in a milk bar/trough. Examples of disinfectants used include acid rinses, iodine soaks, and chlorinated detergents.

### Calf Starter Feed

The types of calf starter fed at these operations consisted of pelletized feed (40%), texturized feed (47%), or a TMR (13%). Pelletized feed was defined as feed where all ingredients were combined into a pelleted form. Texturized feed consisted of feed that included a pellet plus roughage. A TMR was a blend of grain, roughage, liquid, feed additive, etc. Most operations (60%) reported the number of times starter was fed changes over time, ranging between 1 to 5 times a day. All but one operation fed starter to calves ad libitum, and the remaining operation increased the amount of starter fed from 0.5 pounds to 14 pounds over 14 wk. Most operations (93%) reported dumping old feed from buckets. Of the 14 operations dumping old feed, 29% would dump feed as needed, 29% did so twice per week, 7% daily, 7% every 2 wk, 14% based on the weather, 7% based on illness, and 7% reported that whether they dumped feed changed seasonally. For diet formulation, the specific ingredients included can be seen in Table 1. For pelleted feeds, if the presence of roughage and liquids were reported, they were included within the actual pellet. For cleaning of feed buckets in the hutches/pens, 1 ranch cleaned buckets as needed, 10 cleaned buckets between groups of calves, and 4 cleaned buckets every 2 or 3 d.

**Table 1.** Ingredients used in starter diets reported as percentage of operations (n = 15) that use each ingredient. The starter diet is the first diet fed to calves upon or shortly following arrival to the calf ranch

Ingredient	Count	% of Operations
**Grains**		
Cracked corn	2	13.3%
Rolled corn	1	6.7%
Steam-flaked corn	3	20.0%
Whole corn	3	20.0%
Proprietary/Unknown	6	40.0%
Total	15	100.0%
**Roughage**		
Cotton seed hulls	2	13.3%
Ground alfalfa	2	13.3%
Ground long-stem roughage	1	6.7%
Hay	1	6.7%
Proprietary/Unknown	6	40.0%
N/A	3	20.0%
Total	15	100.0%
**Liquids**		
Molasses	10	66.7%
Molasses; Fat	3	20.0%
Molasses; Soybean oil	1	6.7%
Molasses; Water; Agridyne Mix 30	1	6.7%
Total	15	100.0%
**Feed Additives**		
Bovamine	1	6.7%
Calf Insure	1	6.7%
Mineral	1	6.7%
Rumensin	1	6.7%
Bovamine and Rumensin	1	6.7%
Proprietary/Unknown	1	6.7%
N/A	9	60.0%
Total	15	100.0%

N/A: This ingredient was not used in the starter diet.

### Water Offerings

Water was first offered to calves upon arrival at 73% of the operations, 1 d following arrival at 20%, and 2 d following arrival at 7%. In operations housing pre-weaned calves individually (n = 14), water was offered in buckets at most operations (86%), in automatic drinkers at 7%, and through water lines to each pen with nipples in 7% of operations. The operation that housed pre-weaned calves in group pens provided water in an open trough. The waterers in the pre-weaning phase were cleaned daily in 2 of the operations, twice per week in 4, weekly in 2, and between groups of calves in 7 of the operations. For post-weaning group housing, 36% (6/14) offered water in big open troughs and 64% (9/14) used automatic drinkers. In the group pens, waterers were cleaned daily in 2 of the operations, twice per week in 1, weekly in 7, between groups of calves in 3, and seasonally-dependent where they were washed twice per week in the summer and weekly in the winter at 1 of the operations. There is one operation that does not house calves post-weaning thus this question was N/A.

### Weaning Protocol

**Figure 2.**
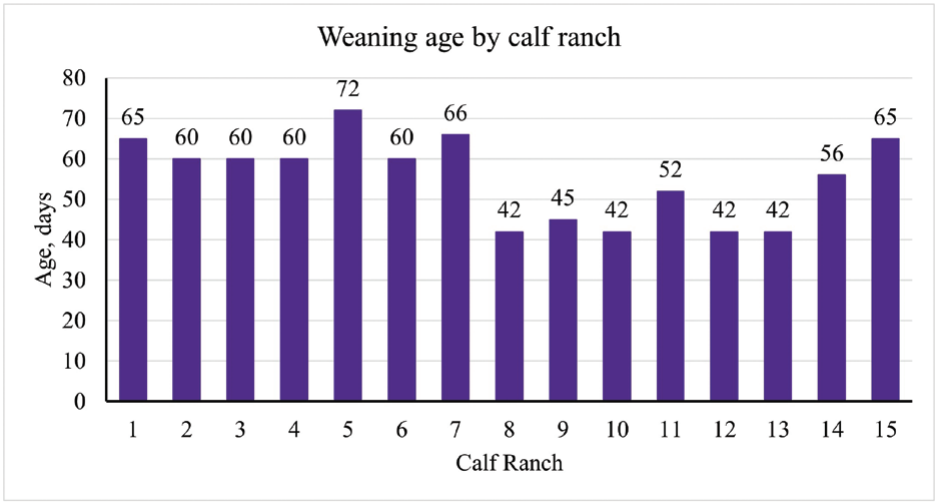
Average weaning (complete removal from milk) age by calf ranch.

For average age completely weaned off milk, 33% weaned calves between 40 and 49 d of age, 13% weaned between 50 to 59 d, 47% between 60 and 69 d, and 7% weaned calves that were greater than 70 d of age. The distribution of weaning age by calf ranch can be seen in Figure 2. All operations used age as the main criteria for making weaning decisions, however, some operations used other criteria in addition to age. For example, 20% of the operations used feed intake as well, 33% considered size of the calf, and 7% took capacity into consideration when making weaning decisions. Most operations (73%) did not have any reason for weaning calves at an earlier age than their cohort, but those that did reported reasons such as capacity, cattle flow, size of the calf, or a combination of capacity, weight, and body condition score. In contrast, fewer operations (33%) reported they did not have any reason to wean a calf at an older age than its cohort, but those that did reported size of the calf, feed intake, cattle flow, capacity, weight, and/or BCS as reasons to wean calves at an older age.

### Movement to and Management of Group Pens Post-weaning

**Figure 3.**
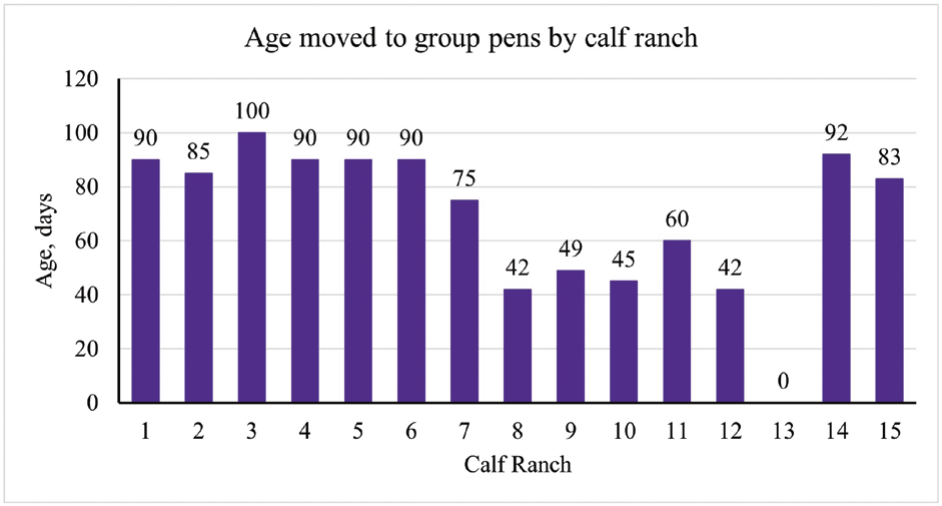
The age of calves when moved to a group pen. Ranch 13 shipped calves to a different facility following weaning.

**Figure 4.**
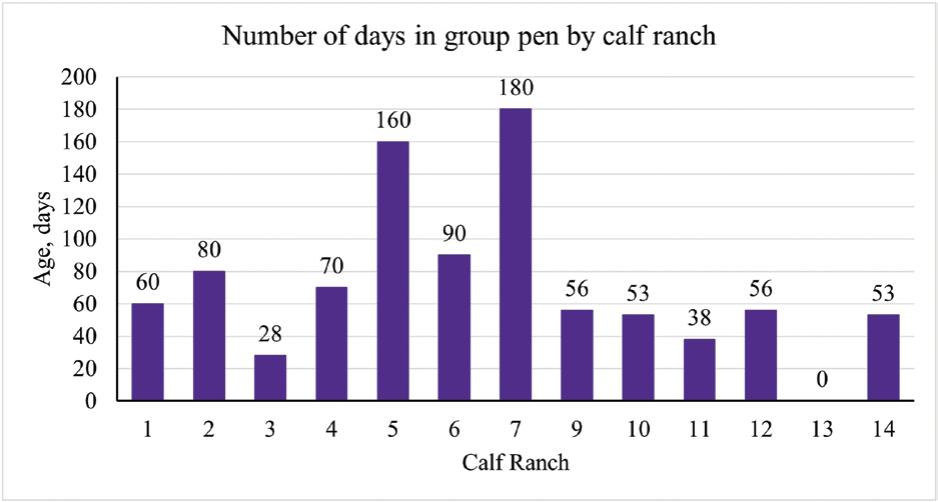
The number of days calves spent residing in a group pen following weaning Ranch 8 shipped calves to another facility following weaning, however the authors were still able to collect information about management of the group pens due to the interviewee’s relation to the owner of the facility. Ranch 13 shipped calves to a different facility following weaning of unknown ownership. Ranch 15 was excluded as they shipped calves based on weight, not days of age. Therefore, they could not specify how long calves resided in a group pen.

There was one operation that did not move calves into a group pen post-weaning as they shipped calves immediately following the weaning period, therefore results from this section are in reference to 14 of the operations. When transitioning calves into group pens post-weaning, 29% of operations moved calves into group pens between 40 to 59 d of age, 21% moved calves between 60 to 79 d of age, 43% moved calves between 80 to 99 d, and 7% moved calves at an age greater than 100 d as seen in Figure 3. Of the 14 operations that transitioned calves to group pens post-weaning, common reasons that calves may be moved to group pens at an earlier age included capacity (14%), size of calf (7%), a combination of the two (7%), or no reason would influence the operation to transition calves to a group pen earlier (71%). Common reasons for moving calves to a group pen at a later age included capacity (14%), cattle flow (14%), feed intake (7%), health status (14%), size of the calf (21%), a combination of size of the calf and feed intake (7%), or no reason would persuade the operation to deter from the age they usually transition calves (21%). Figure 4 shows the total number of days calves resided in a group pen setting for each operation.

Most operations (64%) moved their calves into one group pen where they resided until they shipped, and the remainder moved calves through multiple group pens. Of the 5 operations that transitioned their calves through multiple pens, one operation moved calves twice while four of the operations moved calves three times through group pens with various pen dimensions and head counts. For operations that moved calves to one group pen where they stayed until shipping, 1 ranch placed calves in pens with less than 50 animals, 3 placed them in pens of 50 to 100 animals, 2 placed them in pens of 101 to 150 animals, 2 placed them in pens with 151 to 200 animals, and the remaining operation placed them in a pen with greater than 200 head. For operations that moved calves through multiple group pens, all the first group pens were 25 animals or less. For the second group pen, 40% (2/5) moved the calves to pens of 40 to 50 animals, 40% (2/5) moved them to pens of 51 to 100 animals, and 20% (1/5) moved calves to a pen of more than 100 animals. Four operations moved calves to a third group pen, where 1 operation housed 40 calves together, 2 operations housed 75 to 80 calves together, and the final operation moved calves to a pen of 120 animals. Most operations (79%) reported that they did sort calves into group pens based on specific criteria. Of the operations that sorted into group pens, 1 operation sorted based on breed alone, 2 operations sorted based on health status, 1 sorted based on owner of the calves, 1 sorted based on the location of the pre-weaning housing, 1 sorted based on where calves were going to ship to after the calf ranch, and the remaining 5 operations sorted based on different combinations of the following criteria: sex, origin, breed, weight, age, and genetics.

For bedding of group pens, 79% reported they do bed year-round, 14% do not, and 7% bed seasonally. Of the 12 operations that do provide bedding, 8% use corn fodder, 17% use corn stalks, 17% use shavings, and 58% use straw. The flooring of various group pens consisted of compacted dirt/gravel (7%), a concrete pad (21%), dirt (14%), or dirt with concrete aprons around the bunks and water sources (57%). For pen enrichment, 50% (7/14) reported they do offer some type of enrichment within the group pens. Of these 7 operations, 71% provided lick tubs or salt blocks within the pens, 14% provided tubs of sand, and 14% provided scratching posts. The majority of participants (79%) reported that group pens were cleaned between groups of calves, 7% cleaned pens as needed, 7% weekly, and the remaining 7% cleaned group pens every 2 mo. Group pens were cleaned through scraping (64%), using pitch forks (7%), scraping followed by disinfection of the pen (14%), or unspecified means of cleaning (14%). For bunk cleanliness, 7% cleaned bunks as needed, 21% cleaned bunks daily, 14% did so twice per week, 21% weekly, 7% monthly, and 29% did so between groups of calves.

### Transition/Grower Feed

There was one operation that shipped calves immediately following weaning, so that operation did not answer the questions for this section. The type of transition feed at these operations were described as a TMR (57%), texturized feed (14%), or a pelletized feed (14%). The remaining 14% consisted of 2 operations that fed starter until they shipped calves. One operation shipped calves at 42 d of age, and the second operation fed calf starter in the individual pens as well as group housing until calves shipped at 98 d of age. Of the 12 operations that fed a transition diet, the majority of operations (58%) fed one transition diet, 25% fed two different transition diets, 8% fed three different transition diets, and 8% fed a diet with the same ingredients but varying levels of protein throughout the post-weaning phase. The ingredients used for the transition diets can be seen in Table 2. Five of the operations (36%) fed the transition feed just once a day, 4 fed twice a day, 1 fed three times a day, 1 fed six times a day, and 1 ranch fed as needed with an automatic feed delivery system. The remaining 2 operations fed starter feed in the group pens where one operation fed it in bulk and the other fed it biweekly. The majority of operations (64%) fed ad libitum in group pens, 29% called bunks, and 7% fed an amount between 14 and 16 pounds. A large portion of the participants (79%) reported cleaning old feed from bunks, and 21% did not. Of the 11 operations cleaning feed out of bunks, 35% did so as needed, 18% did so daily, 9% biweekly, 27% weekly, and the remaining 9% did so depending on the weather. Most operations (86%) did not top-dress the feed in group pens. Two operations top-dressed where one operation added antibiotics for the 1^st^ 3 d in the group pen and the other operation fed a coccidiostat for the 1^st^ week within the group pen.

**Table 2.** Ingredients used in transition diets reported as percentage of operations (n = 15) that use each ingredient within the transition diet. Transition diets can be defined as the diet calves are fed after the starter diet in the post-weaning period

Ingredient	Count	% of Operations
**Grains**		
Ground corn	1	6.7%
Steam-flaked corn	8	53.3%
Whole and steam-flaked corn	1	6.7%
Proprietary/Unknown	2	13.3%
N/A*	3	20.0%
Total	15	100.0%
**Roughage**		
Alfalfa	4	26.7%
Corn silage	2	13.3%
Chopped hay	1	6.7%
Alfalfa and cotton seed hulls	1	6.7%
Alfalfa and triticale	1	6.7%
Proprietary/Unknown	2	13.3%
N/A*	4	26.7%
Total	15	100.0%
**Liquids**		
Molasses	6	40.0%
Molasses; Fat	4	26.7%
Molasses; Water; Agridyne	1	6.7%
N/A^†^	4	
Total	15	100.0%
**Feed Additives**		
Bovamine	1	6.7%
Lasalocid	1	6.7%
Vitamins and trace minerals (VTM)	2	13.3%
Rumensin	2	13.3%
Bovamine and Rumensin	1	6.7%
VTM and lactobacillus	1	6.7%
N/A^†^	7	46.7%
Total	15	100.0%

N/A*: These are the operations that fed the starter diet for the entire duration of calf’s time at the faciltiy.

N/A^†^: Three of these operations fed the starter diet for the entire duration of calf’s time at the facility. The remaining number did not use this ingredient in the transition diet.

### Health Challenges and Vaccine/Treatment Protocol

The majority of operations (67%) reported that they do not measure serum total protein upon arrival, and of the 5 operations that did measure serum total protein, 3 of them reported the total protein measures back to the dairies they received calves from, 1 used the total protein measurement to build a separate health protocol for high-risk dairies, and 1 used the total protein measurement as verification of calves receiving sufficient colostrum before arriving to the calf ranch. For vaccination protocols, 6 of the ranches used the same consulting veterinarian and all use the same protocol. Due to this, the answers from those operations were counted as 1 rather than 6 observations. The count and percentage of operations that administered different types of health products upon arrival and during pre-weaning is shown in Table 3. The ranches that administered a toxoid, antiparasitic and an implant during the pre-weaning period were all located in the Midwest and used the same consulting veterinarian. The number of products given to a calf in the pre-weaning phase ranged from a minimum of 3 products to 15 products among operations. For the post-weaning phase, the number of products administered ranged from 0 to 6 products. Most operations (80%) reported that their use of vaccines does not vary, and for those that do, cost of the products and disease outbreak were the reasons reported for changing their health protocols.

**Table 3.** Health products administered at arrival and/or in the pre-weaning period reported as % of operation (n = 10) that gave a product of that category. The 6 operations from the Midwest were counted as 1 response because they all used the same consulting veterinarian and as a result used the same health protocols

Type of product	Arrival	Pre-weaning
Viral respiratory vaccine	60.0% (6/10)	100.0% (10/10)
Clostridial vaccine	0.0% (0/10)	40.0% (4/10)
Bacterial respiratory vaccine	20.0% (2/10)	100.0% (10/10)
Autogenous vaccine	10.0% (1/10)	60.0% (6/10)
Metaphylaxis	0.0% (0/10)	10.0% (1/10)
Antiparasitic	0.0% (0/10)	10.0% (1/10)
Iron supplementation	0.0% (0/10)	10.0% (1/10)
Meloxicam	0.0% (0/10)	10.0% (1/10)

For treatment of respiratory disease, 73% (11/15) of the participants used an antimicrobial, and 27% (4/15) used an antimicrobial and an anti-inflammatory drug. When treating digestive diseases, there was one operation that treated by giving only IV fluids, while one operation treated digestive disease exclusively with electrolytes, and 12 others treated with electrolytes combined with another therapy. Additional therapies and the percentage of operations that use those to treat respiratory and digestive disease can be seen in Tables 4 and 5, respectively. For example, approximately 46.7% of operations treated digestive disease with an antimicrobial. Most of the operations (80%) reported that they do keep individual treatment records. Two of the operations that did not keep treatment records were located in the High Plains and the third was in the Midwest.

**Table 4.** Health products administered to treat respiratory disease

Type of product	Count	% of Operations
Antimicrobial	15	100.0%
Anti-inflammatory drug	4	26.7%

**Table 5.** Health products administered to treat digestive disease reported as percentage of operations that use each product

Type of product	Count	% of Operations
Electrolytes	12	80.0%
IV fluids	9	60.0%
Antimicrobial	7	46.7%
Anti-inflammatory drug	2	13.3%
Peptobismal	2	13.3%
Amino acids	1	6.7%
Serum antibodies	1	6.7%
Charcoal	1	6.7%
Probiotics	1	6.7%
Dextrose	1	6.7%
B vitamins	1	6.7%

The final questions for the calf health section of the survey related to precautions taken during winter storms or heat events. The majority of operations (7/15) added extra bedding for winter storms, 1 added an antimicrobial to the water, 1 closed hutches, 1 built windbreaks, 2 built windbreaks and provided extra bedding, 1 provided heaters, extra bedding, and extra feed, and the remaining 2 ranches did not take any precautions for winter weather events. During heat events, most (8/15) operations took no precautions while the remainder (7/15) took precautions such as adding an antimicrobial to the water, closing hutches, turning on fans, spraying cattle with water, providing shade and/or ensuring waterers stay full.

## DISCUSSION

The results from this survey were recorded from a population selected by convenience sampling. Therefore, the findings should be interpreted with care and may not be representative of all calf raising facilities within the U.S.

The authors’ goal was to describe the management of beef-on-dairy calves at commercial calf ranches since little information has been published in this area. Because of this, broad questions were asked to assess how practices may vary among operations. There have been several national surveys conducted to collect information about replacement dairy heifer calves (Wolf, 2003; USDA, 2010, 2012, 2021; Walker et al., 2012). In those previous surveys, the population consisted of the East (Indiana, Iowa, Kentucky, Michigan, Minnesota, Missouri, New York, Ohio, Pennsylvania, Vermont, Virginia, and Wisconsin) and the West (Arizona, California, Colorado, Idaho, Kansas, New Mexico, North Dakota, Texas, and Washington) (USDA, 2012). In comparison, the present data consisted of ranches from California (n = 2), Kansas (n = 4), New Mexico (n = 1), Texas (n = 2), Indiana (n = 4) and Ohio (n = 2). The herd sizes defined in previous surveys consisted of small (20 to 99 head), medium (100 to 999 head), and large (1,000 or more) whereas in the current survey the sizes were categorized as less than 1,000 (n = 6), 1,000 to 20,000 (n = 2), 20,000 to 50,000 (n = 3), and greater than 50,000 calves (n = 4) (USDA, 2010: 200, 2012).

In the present data, none of the calf ranches solely owned the calves they raise. At 60% of the operations, the dairy of origin retained ownership of the calves, and the remaining 40% was a combination of dairy-owned and calf ranch-owned calves. In 3 surveys conducted asking farmers about their use of beef semen on dairy farms, the majority of respondents in each survey reported marketing their calves prior to the finishing phase (Halfman and Sterry, 2019; Pereira et al., 2022; Felix et al., 2023). One of the surveys was conducted in Wisconsin, Michigan, and Iowa, and 32 of the 45 farms marketed beef-on-dairy calves at a week old or less, and 9 of the farms retained ownership and marketed them as finished cattle (Halfman and Sterry, 2019). Similarly, the majority of surveyed dairy farms in Pennsylvania and the northeast (CT, DE, ME, MD, MA, NH, NY, RI, and VT) (89%; 398/448 farms) reported that they did not retain ownership and marketed calves before the finishing phase (Felix et al., 2023). This information contrasts what the participants reported in the present study. A difference to note is the location of the calf ranches surveyed in the cited surveys versus the operations from this dataset.

The NAHMS Dairy 2007 and Dairy 2011 studies reported the average age of arrival for preweaned heifers was 3.4 d, and similarly, most operations (n = 10) from the present survey reported receiving calves between 2 to 4 d of age (USDA, 2010, 2012). Walker et al. reported the median age of arrival for calves to heifer raising facilities and mixed heifer and bull calf raising operations was 2 to 3 d (Walker et al., 2012). However, it is important to note that in previous surveys, heifer raising facilities report receiving preweaned as well as weaned heifers (Wolf, 2003; USDA, 2010, 2012). In 2003, Wolf reported the most common age heifers entered an off-site “custom dairy heifer grower” was following weaning, after 2 mo of age and before 6 mo of age (Wolf, 2003). In previous surveys, dairy replacement heifer raising facilities received both pre-weaned and weaned heifers, whereas all calf ranches in the present data only received pre-weaned calves. The calf ranches described in this survey raised calves from a very young age and raised them through weaning at least. As such, a shift can be seen in heifer growing facilities raising calves off-site from weaning as described in Wolf (2003) to calf ranches raising dairy as well as beef-on-dairy calves from as young as a day old as reported by NAHMS and the present data (Wolf, 2003; USDA, 2010, 2012).

For these young calves, pre-weaned housing consisted of individual hutches outdoors at 9 operations, individual pens inside barns at 5 operations, and group pens inside a barn at 1 operation. In the 2011 NAHMS survey, there was a difference in housing of preweaned heifers based on region with more than 90% of operations in the West region (AZ, CA, CO, ID, KS, NM, ND, TX, WA) using individual hutches/pens outdoors, 42% of eastern operations (IN, IA, KY, MI, MN, MO, NY, OH, PA, VT, VA, WI) using individual pens inside barns, and only 30% of operations in the east using outdoor hutches/pens. There was 19.4% of eastern operations that housed preweaned heifers in a group pen inside a barn (USDA, 2012). These data are similar to the present as the 6 operations that housed preweaned calves indoors, in individual pens or group pens, were located in the Midwest (OH and IN). Although the present data represents a significantly smaller number of operations than those described by previous surveys, the similarities found among regions show how housing choices could be dependent on the climates of different parts of the country. It would be interesting to know how housing varies based on the size of operation; however, the NAHMS data did not report types of housing based on herd size, only region. Thus, the authors cannot speculate on potential housing differences depending on the capacity of the operation.

When asked about type of milk fed, previous surveys reported that approximately 86% of all operations fed any milk replacer to preweaned heifers which is reflected in the present findings where 93% of participants fed milk replacer in some amount (USDA, 2012). There have been many studies investigating different milk feeding protocols related to milk allowance, milk feeding frequency, and feeding methods. Welk et al. performed a systematic review with a total of 69 articles and found increasing milk allowance can improve growth during preweaning and the use of gradual weaning methods can facilitate starter intake and allow calves fed higher amounts of milk to maintain growth advantages postweaning (Welk et al., 2023). The milk feeding protocols reported in the current survey were variable across operations. All operations fed milk twice per day upon arrival, however the amount of milk fed varied between 2 and 3 quarts per feeding among operations. Most operations (87%) reported gradually weaning calves off milk in the present survey. In the 2011 Dairy Heifer Raiser survey, 70% of all operations fed between 4 and 5 quarts of milk per day and 93% fed milk twice a day (USDA, 2012). However, it’s difficult to make comparisons between the present and past data as the NAHMS survey did not describe potential changes in milk feeding practices over time. The primary equipment used to feed milk or milk replacer reported by NAHMS was buckets, whereas most operations in this survey used bottles. Thus, there were consistencies among operations in the present survey for milk feeding protocols upon arrival, but changes in frequency of feedings and amount fed as the calves aged.

According to the NAHMS 2011 heifer raising survey, the average age at which calves received water and calf starter was 6 d (USDA, 2012). In the present survey, water and calf starter was offered within 2 d of arrival at all operations, with the majority offering both upon arrival. The NAHMS survey data from 2011 did not report about the type of starter feed, ingredients, amount, or frequency of starter fed. Ghaffari et al. performed a comprehensive review and meta-analysis of studies investigating different forms of calf starter (ground, pelleted, textured, ground diets blended with hay, and textured diets blended with hay) and their effects on starter intake of dairy calves (Ghaffari and Kertz, 2021). They concluded that feeding textured starter feed compared to pelleted feed resulted in greater intake levels, but there was not sufficient evidence for a recommended starter physical form due to the variation among calf studies (Ghaffari and Kertz, 2021). The grain sources for the studies included in the meta-analysis consisted of corn, barley, oats, and/or wheat middlings at various levels. The results from the present data described the ingredients used in starter feeds, however, the inclusion rate was not collected in this survey. The grains used consisted of cracked, rolled, steam-flaked, whole corn, or were proprietary. While the present data provides more information about the type of diet commercial calf ranches are feeding, it seems there is not a consistent practice across the industry for starter diets fed to pre-weaned calves.

For weaning age, traditionally raised beef calves are weaned at approximately 195 d (USDA, 2020). In heifer raising facilities, the average weaning age was 7 wk, or about 45 to 50 d (USDA, 2012; Walker et al., 2012). In the present data, 54% of operations raising crossbred beef-on-dairy calves weaned at an age between 60 and 75 d, 13% weaned between 50 to 59 d, and 33% weaned at less than 50 d of age. The difference in weaning age between replacement dairy heifers and beef-on-dairy calves may be attributed to differences in production plans. Dairy replacement heifers are typically sent to off-site facilities and later return to the dairy of origin, with less emphasis on weight at weaning. In contrast, beef-on-dairy calves are generally sent to a feedlot or backgrounding facility prior to the feedlot. In this scenario, the weight at which the calves leave the facility is a key factor if calves are marketed on a weight basis. However, for dairy replacement heifers, body weight is less critical, as they are retained within their own dairy system and not typically sold to external buyers. This could be a reason for the difference seen in weaning age among these breeds of calves.

Region might also play a role in difference of weaning age as the 2011 NAHMS survey reported that weaning age was generally higher in the West region (8.9 wk, 62 d) compared to the east (6.7 wk, 47 d) (USDA, 2012). Similar results were reported in the present data where calves were weaned at a younger age in the Midwest (42 to 52 d) compared to the other regions (High Plains, 60 to 72 d; West, 56 to 65 d), however this data is confounded by operation size and housing type. All operations located in the Midwest had a total capacity of less than 1,000 calves and housed their calves individually within pens inside a barn. Thus, it is difficult to understand how region truly plays a role in weaning age due to potential confounding variables.

Most of the operations housed weaned calves in group dry lot pens. In dairy heifer raisers, less than 20% housed multiple weaned heifers in dry lot pens, 16% used freestall housing, 20% used bedded pack/open shed housing, and 15% housed multiple heifers in an pen inside a barn (USDA, 2012). Therefore, the authors found little to no information that confirms the findings of this survey in relation to beef-on-dairy calves. There was also little reported data regarding group housing for dairy heifer raising facilities; however, the management of weaned calves within these group pens mimics what is common practice for backgrounding beef calves in confined dry lot pens. Backgrounding is the phase between weaning and shipment to a feedlot for finishing where calves can acclimate to eating from bunks in a group setting. All operations included in this survey did house calves in a group setting post-weaning except for one. This operation housed calves in a group pen pre-weaning and shipped calves somewhere else immediately following weaning. There was variation among the operations on how they transitioned calves to group pens in the size of the pens, head count of pens, and whether calves were moved between multiple group pens. Therefore, it is difficult to identify any kind of pattern or potential industry standard among the surveyed operations.

There was also little to no published literature about the diet fed to calves in group pen settings following weaning. Machado and Ballou (2022) found in their literature review that most producers allow calves to continue eating calf starter for approximately a month after moving into group pens before switching them to a “transition diet.” Transition diets are typically TMRs that mimic the receiving diet cattle will be fed upon arrival to a feedlot. A meta-analysis of trials in grower Holstein calves was conducted to evaluate the level of starch concentration on growth and performance which demonstrates that research regarding formulation of post-weaning diets has been conducted (Hu et al., 2018). However, there is no survey data that describes post-weaning feed protocols in the commercial calf ranch or dairy heifer raising facility. The present data revealed inconsistencies among operations in the number of times fed per day, the amount fed, and the transition between multiple diets post-weaning. Feed delivery varied from once per day to as many as 6 times per day to automatic feed delivery as needed. The majority of operations fed ad libitum but there were 4 operations that managed quantity fed through calling bunks. Five operations had feeding programs that consisted of multiple diets that calves transitioned to as they grew older. Eight operations fed a TMR, similar to that of a backgrounding facility or a feedlot, and 6 fed either a pellet or a texturized feed consisting of a pellet and roughage. Five of these 6 were located in the Midwest. More research is required to fully understand how the formulation of and transition from calf starter to a transition/grower diet affects calf performance. There are currently no peer-reviewed recommendations for formulating transition diets for commercial calf ranches that the authors found. Therefore, it is assumed that operations feeding a TMR are formulating the diet like they would a receiving diet found at a feedlot, however, there is no current literature comparing these types diets.

The health proportion of this survey showed that most operations (10/15) did not routinely measure total protein upon arrival to the calf ranch. Similarly, NAHMS reported that 60% of all operations did not measure serum proteins (USDA, 2012). When asked about treatment protocols for respiratory disease all operations treated with an antimicrobial, and a portion (4/15) administered an anti-inflammatory in addition to the antimicrobial (Table 4). Treating respiratory disease with an antimicrobial is standard practice within any sector of the cattle production system. For treatment of digestive diseases, 53% of operations treated with electrolytes in combination with another type of treatment. Many operations used IV fluids in addition to electrolytes (Table 5). Treatment of digestive diseases in calves, specifically scours, typically focuses on rehydrating the calf (Stoltenow and Vincent, 2003; Spence, 2006; Van Emon, 2024). However, the Dairy Heifer Raiser survey from 2011 did report that antibiotics were used to treat diarrhea in preweaned heifer on 85.7% of operations (USDA, 2012). This is interesting as less than 60% of operations in the present data reported use of an antimicrobial to treat digestive disease. This slight decrease in the use of antimicrobials could be due to the rising concern of antimicrobial resistance among producers and consumers; however this cannot be confirmed with the data collected.

## LIMITATIONS

A limitation of this study includes the sample size. The calf ranches that participated were identified and selected through convenience sampling. There were only 15 calf ranches that participated in this survey; however, the authors believe the results still provide valuable information about the management of beef-on-dairy calves due to the knowledge gap in published literature. It is also important to acknowledge that the health/vaccine protocol results from this survey were confounded by use of the same consulting veterinarian, specifically in the Midwest region. Other confounding variables include the region the operation was located.

## CONCLUSIONS

The information provided by this survey is valuable as it provides insight to the management of beef-on-dairy calves within commercial calf ranches. As the number of beef-on-dairy calves increases, the need for more research on their raising, performance, growth, and health is pertinent. This research highlighted the knowledge gap surrounding the management of beef-on-dairy calves by identifying previous surveys conducted with dairy heifer raising facilities and describing management practices used at commercial calf ranches albeit in a small sample (n = 15). There has been speculation that beef-on-dairy calves raised alongside dairy heifers are managed differently, however the management practices of beef-on-dairy calves had not been previously described until now. Therefore, it is important to ask the industry these questions and determine the practices actually employed by calf ranches.
